# Evaluation of a Health Information Technology–Enabled Panel Management Platform to Improve Anticoagulation Control in a Low-Income Patient Population: Protocol for a Quasi-Experimental Design

**DOI:** 10.2196/13835

**Published:** 2020-01-13

**Authors:** Valy Fontil, Dhruv Kazi, Roy Cherian, Shin-Yu Lee, Urmimala Sarkar

**Affiliations:** 1 Center for Vulnerable Populations University of California San Francisco, CA United States; 2 Smith Center for Outcomes Research in Cardiology Beth Israel Deaconess Medical Center Harvard University Boston, MA United States; 3 Outpatient Pharmacy Zuckerberg San Francisco General Hospital San Francisco, CA United States

**Keywords:** population health, warfarin, biomedical technology

## Abstract

**Background:**

Warfarin is one of the most commonly prescribed medications in the United States, and it causes a significant proportion of adverse drug events. Patients taking warfarin fall outside of the recommended therapeutic range 30% of the time, largely because of inadequate laboratory monitoring and dose adjustment. This leads to an increased risk of blood clots or bleeding events. We propose a comparative effectiveness study to examine whether a technology-enabled anticoagulation management program can improve long-term clinical outcomes compared with usual care.

**Objective:**

Our proposed intervention is the implementation of an electronic dashboard (integrated into a preexisting electronic health record) and standardized workflow to track patients’ laboratory results, identify patients requiring follow-up, and facilitate the use of a validated nomogram for dose adjustment. The primary outcome of this study is the time in therapeutic range (TTR) at 6 months post intervention (a validated metric of anticoagulation quality among patients receiving warfarin).

**Methods:**

We will employ a pre-post quasi-experimental design with a nonequivalent usual-care comparison site and a difference-in-differences approach to compare the effectiveness of a technology-enabled anticoagulation management program compared with usual care at a large university-affiliated safety-net clinic.

**Results:**

We used a commercially available health information technology (HIT) platform to host a registry of patients on warfarin therapy and create the electronic dashboard for panel management. We developed the intervention with, and for, frontline clinician users, using principles of human-centered design. This study is funded until September 2020 and is approved by the University of California, San Francisco Institutional Review Board until June 22, 2020. We completed data collection in September 2019 and expect to complete our proposed analyses by February 2020.

**Conclusions:**

We anticipate that the intervention will increase TTR among patients taking warfarin and that the use of this HIT platform will facilitate tracking and monitoring of patients on warfarin, which could enable outreach to those overdue for visits or laboratory monitoring. We will use these findings to iteratively improve the platform in preparation for a larger, multiple-site, pragmatic clinical trial. If successful, our study will demonstrate the integration of HIT platforms into existing electronic health records to improve patient care in real-world clinical settings.

**International Registered Report Identifier (IRRID):**

DERR1-10.2196/13835

## Introduction

### Background

Warfarin is one of the most commonly prescribed medications in the United States, with more than 20 million Americans taking warfarin anticoagulation therapy to prevent the formation of blood clots. As one of the most common culprits of adverse drug events (ADEs) in outpatient settings [1], warfarin dosing must be individualized to be effective; overdosing of warfarin can cause serious bleeding complications, whereas underdosing does not provide adequate protection against thromboembolism. As such, the National Action Plan for ADE Prevention targets blood thinners as a high priority for intervention [2].

Despite more than 50 years of clinical experience, studies suggest that patients taking warfarin fall outside of the recommended therapeutic range 30% of the time [3]. Therapeutic range is based on a laboratory test called the international normalized ratio (INR), and a patient’s time in therapeutic range (TTR) is associated with lower risk of developing blood clots from underdosing of warfarin or bleeding events from overdosing [4]. Therefore, warfarin treatment requires periodic monitoring with blood tests to inform dose adjustment [5].

Unfortunately, solutions for efficient monitoring of warfarin treatment are still lacking, especially in settings such as safety-net clinics with relatively limited resources and health technology infrastructure. In safety-net clinics caring for low-income, uninsured, or underinsured patients, maintaining warfarin in the therapeutic range poses an additional challenge because of limited health literacy and educational attainment for a large proportion of the patient population [6,7], prevalent impairment in cognitive function [8], and various socioeconomic challenges [9]. Hence, proactive and efficient strategies that support outpatient warfarin monitoring are urgently needed.

As anticoagulation therapy relies on well-established, standardized care protocols with clear safety and efficacy targets (eg, INR monitoring), it is well suited to health information technology (HIT) approaches that can identify patients who need a specific treatment, monitoring, or intervention and help health care providers execute the appropriate management plan. These software platforms have been widely used for outreach and management of chronic illnesses, including diabetes, asthma, cancer, depression, and congestive heart failure [10-14], where their use improves health outcomes and is cost-effective [15-17]. However, many HIT interventions are not adequately integrated with current electronic health records (EHRs), forcing health systems to add layers of complexity to clinician workflow that are seldom feasible in safety-net clinics with limited resources [18]. Moreover, current platforms are not customized for anticoagulation therapy to facilitate the attainment and tracking of safety and efficacy targets. This study aims to resolve this limitation by implementing a locally customized, user-designed HIT tool that interfaces with the EHR and focuses on improving the quality of anticoagulation therapy.

### Objective

In this study, we propose to design and test an electronic dashboard for panel management of patients taking warfarin anticoagulation therapy at a large university-affiliated safety-net clinic. We have previously developed an electronic registry of patients taking warfarin and a team-based workflow for scheduling or rescheduling clinic appointments for patients outside of the therapeutic range [19]. This prior pilot intervention led to an improvement in patient attendance to visits for anticoagulation management [19]. We will use a commercially available HIT platform to host our patient registry and create an electronic dashboard for panel management that will enable providers to track patients’ INR, implement a workflow for scheduling patients to visits, and use a validated electronic anticoagulation treatment nomogram for dose adjustment during the visit.

## Methods

### Study Design

This is a pre-post quasi-experimental design with a nonequivalent usual care comparison site. We will use a difference-in-differences approach to compare the effectiveness of a technology-enabled panel management anticoagulation program with usual care in improving TTR.

### Study Population and Setting

#### Clinical Sites

The site that will undergo the intervention is a large university-affiliated anticoagulation clinic within a network of 12 safety-net clinics in San Francisco that serve a racially and ethnically diverse population of low-income patients. A team of 3 part-time clinical pharmacists, 1 part-time nurse practitioner, a clinical coordinator, and a physician medical director provides care to approximately 250 patients who receive primary care in any of the 12 clinics in the city’s public health network. This network is operated by the San Francisco Department of Public Health (SFDPH), and its member clinics use the same electronic health records. Our comparison site is another anticoagulation clinic site within this network, which is chosen for its similarity with the intervention site with regard to its university affiliation and the demographic composition of its patient population. The comparison site delivers anticoagulation to approximately 50 patients receiving primary care at the family health center; 2 part-time clinical pharmacists and a physician medical director staff this clinic.

As both clinics are within the SFDPH, they follow the same protocols for standard management (monitoring intervals of 4 weeks before establishing therapeutic control and 12 weeks once therapeutic control has been achieved for 3 consecutive visits). The standard management includes a protocol for patients scheduled to undergo high-risk surgical operations. Over the past 3 years, these clinics have sought to use an internal guideline for switching patients from warfarin to direct-acting oral anticoagulants (DOACs) based on the duration of treatment, most recent INR, warfarin medication adherence, drug-drug interactions, indication for anticoagulation, and kidney and liver function.

#### Time Horizon

The period of analysis will include a 12-month preintervention period and a 12-month postintervention period that will each include an *enrollment* period in the first 6 months of pre and postintervention.

#### Enrollment Inclusion Criteria

The study will include all adult patients aged 18 years or above on anticoagulation therapy (such as warfarin, apixaban, rivaroxaban, fondaparinux, dabigatran, and edoxaban) identified by ICD-10 (International Classification of Diseases, tenth revision) Z79.01, who present to anticoagulation clinic for initiation or management of anticoagulation therapy over 2 *enrollment* periods of 6 months at the 2 large academic safety-net clinics (intervention and comparison site) before and after the start of the intervention. We chose the enrollment period of 6 months to enable at least 6 months of follow-up for every patient monitored in the study in both pre and postintervention periods.

#### Intervention

**Figure 1:**
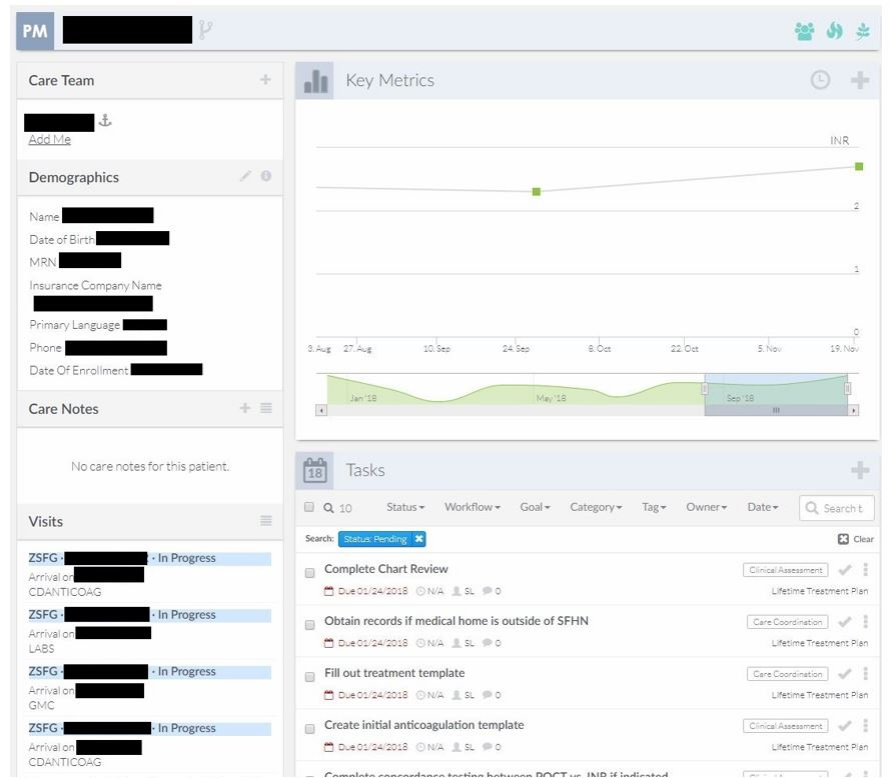
Screenshot of electronic dashboard and panel management interface.

The intervention uses a new electronic dashboard (Figure 1) for panel management of patients on warfarin anticoagulation therapy to improve therapeutic efficacy. The intervention will feature the following elements:

- An electronic dashboard that displays medication information, TTR, and other laboratory results. The dashboard will present relevant data on patients prescribed anticoagulants to facilitate more effective and efficient panel management. The dashboard is customized to show information important for the safe management of these medications (such as current medications, laboratory results, attendance rate to scheduled visits, and language spoken by patients). The dashboard can be used to sort patients according to desired criteria such as diagnoses, specific TTR parameters, or patients with missed scheduled appointments or laboratory monitoring. Finally, the dashboard can be used to create tasks, assign workflow, and send reminders to clinic providers.
- A panel management workflow that will include patient outreach activities, such as appointment reminders and rescheduling of missed appointments; communication from the pharmacist to the patient’s primary care provider or medical specialists, such as alerts to consider novel alternative anticoagulation therapy (eg, DOAC); and suspected or pending adverse effects based on early signs, such as bruising or drug interactions.
- Integration of evidence-based treatment nomogram that will be accessible on the platform for use by the pharmacist to guide therapeutic treatment decisions. Although multiple guidelines addressing anticoagulant management are available to clinical providers, they are not integrated at the point of care. The dashboard will display relevant clinical guidelines while providers are managing their patients. As an example, the warfarin dosing nomogram will be shown on the screen at the same time the providers are managing a patient on warfarin.

#### Electronic Dashboard Development

We developed the dashboard in partnership with our frontline anticoagulation providers using the principles of human-centered design [20], which emphasizes the use of rapid prototyping, early and continuous stakeholder engagement, and iterative development and evaluation of interventions. We partnered with a commercially available health care technology platform (CipherHealth LLC) to host the electronic dashboard [21]. Our study team includes a frontline anticoagulation provider (SL) who conducted informal interviews with other frontline anticoagulation providers at the intervention clinic site to assess their needs and potential usability of the dashboard and an expert cardiologist (DK) who collaborated to create the initial dashboard prototype. We developed subsequent alpha versions of the prototype collaboratively with 2 additional coinvestigators, including an implementation scientist (VF) and a patient safety and digital health technology researcher (US), who are both practicing primary care physicians at the intervention site. We provided the final alpha version to the anticoagulation providers for use over a period of 4 weeks, and we conducted direct observations and informal interviews that led to additional modifications of the platform based on user feedback.

#### Usual Care Comparison

Under current practice (ie, usual care), patients who receive care at either site are referred to an anticoagulation clinic where a pharmacist or nurse practitioner assesses the patient and manages their anticoagulation therapy on an ongoing basis. At each visit, either in person or virtual (by phone), the provider reviews the indication, treatment duration, and relevant history and proactively screens whether a patient qualifies to be transitioned to a DOAC medication. The frequency of follow-up visits is determined by INR results, INR trends, and other clinically relevant factors, including age and bleeding or clotting risks. Current practice does not include panel management or the use of a HIT tool that facilitates panel management and integration of an evidence-based treatment nomogram at the point of care.

### Outcomes and Measurements

#### Primary and Secondary Outcomes

The *primary outcome* will be TTR, a well-established intermediate clinical outcome that is the most widely used metric of anticoagulation quality among patients receiving warfarin [22-25]. It is defined as the proportion of time during which the INR is within the range deemed therapeutic for the indication for which the patient is receiving warfarin therapy. For instance, an INR between 2 and 3 inclusive is therapeutic for patients receiving warfarin for secondary prevention of venous thromboembolism, but an INR between 2.5 and 3.5 inclusive is considered therapeutic for patients with mechanical mitral valves. We will calculate TTR using the Rosendaal method [26], which uses linear interpolation to assign INR values for each day between consecutive INR values. We will calculate the TTR for patients with at least 5 INR laboratory values over the course of at least 6 months. Patients who stop coming to the clinic before or are transitioned to DOACs before this threshold will be censored. The *secondary outcome* will be proportion in therapeutic range defined as the proportion of laboratory INR values that are within the therapeutic range (ie, number of INR values in range divided by the total number of laboratory INR tests performed, regardless of the interval between these values).

#### Exploratory Endpoints

We will examine a number of process measures that are on the pathway to achieving therapeutic goals for anticoagulation, including time from out-of-range INR value to patient contact and time from warfarin initiation to first therapeutic INR (Table 1). We will measure other process-of-care metrics, including (1) patient’s attendance rate to scheduled visits for anticoagulation management, (2) proportion of patients not meeting monitoring guidelines (defined as those who do not have INR testing at least every 56 days based on National Quality Forum recommendations [16]), (3) appropriate duration of therapy (defined as the days of observed warfarin therapy divided by the recommended days of therapy), and (4) provision of telephonic or other remote visits for anticoagulation management. We will also report clinical endpoints such as bleeding complications (hospital visits for bleeding and minor vs major hemorrhagic strokes), incident deep venous thrombosis (DVT), incident pulmonary embolism (PE), and ischemic stroke, although we are likely not adequately powered to detect statistically significant differences between the control and intervention arms. In addition to these process measures for warfarin patients, we will also measure adherence to protocols for screening and transitioning patients to DOACs, which requires assessing patients for medication adherence, drug-drug interactions, and liver and renal function.

**Table 1:** Variables, outcomes, and process measures with definitions.

Variables, outcomes, and process measures		Definition	Rationale
**Independent variables**			
	Basic demographics	Age, gender, race and ethnicity, language, and insurance	Assess patient population
	Diagnoses	Atrial fibrillation, atrial flutter, stroke, DVT^a^, mitral valve replacement, atrial valve replacement, pulmonary arterial hypertension, and PE^b^	Assess prevalence of indications for anticoagulation
	SAMe-TT_2_R_2_ score (sex, age, medical history, treatment, tobacco use, and race)	A clinical scoring system designed to predict which patients on oral vitamin K antagonists (eg, warfarin) will reach an adequate TTR^c^ (>65%-70%)	Assess baseline likelihood of achieving/maintaining anticoagulation control
**Outcomes**			
	TTR	Days in range divided by total days on warfarin	Assess overall treatment efficacy
	Proportion in range	INR^d^ values in range divided by total INR values measured	Secondary measure of treatment efficacy
	TWTR^e^	Days from first administration of warfarin to first therapeutic INR value	Assess efficiency of achieving therapeutic control
**Process measures**			
	Time from out-of-range INR value to patient contact	Days until patient outreach after abnormal INR value	Assess responsiveness of the clinic to abnormal values
	Attendance rate to scheduled visits	Proportion of visits attended (completed visits divided by scheduled visits)	Assess efficiency of clinical operations
	Proportion of patients meeting monitoring guidelines	Proportion of patients who receive regular 56-day monitoring	Assess adherence to treatment guidelines (ie, nomogram)
	Appropriate duration of therapy	Observed duration (days) of anticoagulation therapy divided by recommended total duration	Assess the extent of overtreatment
	Provision of telephonic or other remote visit for anticoagulation management^f^	Proportion of patients transitioned from in-person to phone visits (related to TWTR)	Assess adherence to workflow protocol and overall clinic performance^f^
	DOAC^g^ transitions	Proportion of eligible patients screened and/or transitioned to DOACs	Assess adherence to screening and transition protocols
**Other clinical outcomes**			
	Bleeding complications	Incidence of bleeding during treatment	Assess the incidence of adverse events
	DVT	Incidence of DVT during treatment	Assess the incidence of adverse events
	PE	Incidence of PE during treatment	Assess the incidence of adverse events
	Ischemic stroke	Incidence of stroke during treatment	Assess the incidence of adverse events

^a^DVT: deep venous thrombosis.

^b^PE: pulmonary embolism.

^c^TTR: time in therapeutic range.

^d^INR: international normalized ratio.

^e^TWTR: time from warfarin initiation to first therapeutic INR.

^f^The workflow protocol recommends that patients with international normalized ratio values consistently in range must be transitioned from in-person to telephone visits. If the intervention is effective, we would expect an increase in patients switched to remote telephonic monitoring.

^g^DOAC: direct-acting oral anticoagulant.

#### Independent Variables

We will measure baseline patient demographics such as age, gender, race and ethnicity, and percent poverty in zip code. Other independent variables will include baseline history of chronic kidney disease, ischemic heart disease, stroke, atrial fibrillation, mitral valve replacement (MVR), atrial valve replacement (AVR), DVT, pulmonary arterial hypertension (PAH), and PE.

### Analysis Plan

#### Overview

We will use the difference-in-differences approach to compare the effectiveness of the technology-enabled anticoagulation management program with usual care in improving TTR. Implementation using a linear mixed model (LMM) will allow us to adjust for patient-level confounders; in addition, many patients will provide TTR outcomes both before and after implementation at the intervention clinic and in the matching periods at the control clinic, providing further natural control of confounding. We will also conduct a series of exploratory analyses of secondary outcomes, subgroup analyses to examine heterogeneity of effect, and exploratory mediation analyses to understand the processes by which the intervention might provide greater effectiveness.

#### Difference-in-Differences Analytic Approach

The primary analysis will use an LMM for potentially repeated values of TTR, which are calculated for each patient using the Rosendaal method described above and transformed as needed to meet normality assumptions. The LMM will include a random effect for patient to account for the within-patient correlation of the repeated TTRs and will also include fixed effects for clinical site, intervention period (pre vs post), and their interaction. The LMM coefficient for the interaction between clinical site and intervention period will be interpretable as the difference of differences, the focus of interest. In addition, adjusted mean TTRs by clinic and period will be obtained as linear combinations of the coefficients in the model. To further control confounding, the LMM will include patient-level factors, including baseline gender, race, age, desired value of INR, tobacco use, concurrent use of medications that interfere with warfarin, and presence of a high-risk comorbid condition at baseline (hypertension; diabetes; ischemic heart disease; congestive heart failure; stroke; and pulmonary, hepatic, or renal disease). We have chosen these covariates because they are the known predictive factors for the efficacy of warfarin therapy used to calculate the SAMe-TT_2_R_2_ score (sex, age, medical history, treatment, tobacco use, and race), a widely used clinical prediction rule for achieving and maintaining a desired TTR of greater than 65%, based on common clinical factors that influence INR and anticoagulation control [27]. We define baseline as the time of warfarin initiation for each patient. The model will also adjust for reasons for anticoagulation therapy such as having a baseline diagnosis of atrial fibrillation, MVR, AVR, DVT, PAH, and PE.

#### Analysis of Process Measures

We will employ the difference-in-differences analytic approach described above, using LMMs or generalized LMMs as appropriate to each outcome, to compare pre-post differences between the intervention and usual care clinics in the process outcomes listed in Table 1.

#### Subgroup Analysis to Test for Heterogeneity of Treatment Effects

We will produce strata-specific analyses stratified by the demographic characteristics and test for interactions by intervention arm and demographic subgroups such as age groups, race/ethnicity, and gender.

#### Multiple Hypothesis Testing

We will report the results of the hypothesis test for the primary outcome without adjustment for multiple hypothesis testing. No formal penalization for multiple hypothesis testing is planned for the analysis of process measures or subgroup analyses as these will be treated as exploratory and hypothesis generating. We will report 95% confidence intervals for all our estimates.

#### Multiple Imputation for Missing Data

We do not anticipate any missing data for our primary outcome. In the event of missing data, we will use a multiple imputation strategy to address missing data. In addition to multiple imputation under the standard assumption that data are missing at random (conditional on observed covariates and outcomes), we would also implement sensitivity analyses using imputation under plausible missing not at random scenarios.

#### Data Source, Collection, Management, and Safety

Consistent with the principles of pragmatic clinical trials, we will use EHR data for patient identification and assessment of intervention implementation and outcomes. We have established agreements between CipherHealth and the SFDPH to ensure data security. Data will flow between SFDPH EHRs and CipherHealth through secure file transfer protocols. SFDPH and CipherHealth programmers will regularly review data security procedures. All individuals who have access to patient data are either (1) clinicians with prior access or (2) research staff approved by the University of California, San Francisco internal review board, who have completed training in research ethics and compliance. Identifiable data will only be used for standard patient care and management; all data extracted from the platform for analyses will be deidentified.

#### Minimum Detectable Effects

Preliminary analysis showed that 350 patients would provide 80% power in a test with 2-sided significance level of 5% to detect a difference of differences of 2 to 5 TTR percentage points, based on the proposed analysis using an adjusted LMM. The minimum detectable effect will depend on the intraclass correlation of the repeated TTR measures (assumed range: 0.80-0.95) and the percentage of patients providing measurements in both periods (assumed range: 50%-100%). These calculations assume that the standard deviation of the TTR outcome is 20 percentage points, based on published literature on TTR [28], and these calculations are penalized for covariate adjustment using an adaptation of the variance inflation factor [29].

## Results

We used a commercially available HIT platform to host a registry of patients on warfarin therapy and create an electronic dashboard for panel management. We developed the HIT dashboard interface and co-designed the intervention with, and for, frontline clinician users. This study is funded until September 2020 and is approved by the University of California San Francisco Institutional Review Board until June 22, 2020. We completed data collection as of July 2019 and expect to complete our proposed analyses by February 2020.

## Discussion

We propose a quasi-experimental study to evaluate the implementation of an integrated HIT intervention for management of anticoagulation and examine pre-post outcomes on clinical and implementation outcomes. The HIT platform will facilitate tracking and monitoring of patients on warfarin and enable outreach to those overdue for visits or laboratory monitoring.

We developed the dashboard in partnership with our frontline anticoagulation providers using the principles of human-centered design [20], which emphasizes the use of rapid prototyping, early and continuous stakeholder engagement, and iterative development and evaluation of interventions. An important constraint was its integration into the existing electronic health record with minimal disruption of the existing workflow of anticoagulation service providers. We believe this emphasis on usability is critical to the successful adoption of a HIT intervention.

We anticipate that the intervention will facilitate tracking and monitoring of patients on warfarin, allowing providers to reach out to those with abnormal results (who may need dose adjustments) as well as those overdue for visits or laboratory monitoring. Coupled with increased use of validated nomograms for dose adjustment (which has been previously shown to be a key predictor of improved TTR), our technology-enabled panel management program will increase the proportion of time in TTR among patients taking warfarin. Insights from this study will help us improve and adapt our HIT platform in preparation for a larger, pragmatic clinical trial powered for clinical outcomes. Such a trial would also examine the cost-effectiveness of the intervention.

This project is innovative with significant implications for use of HIT to improve clinical care. First, use of an HIT-enabled intervention to improve anticoagulation management in resource-constrained settings, such as safety-net clinics, could improve outcomes and reduce adverse events at the national level, as these clinics often provide care for a disproportionate share of high-risk and high-cost patients. A common complaint of many widely used electronic health record systems is the relative lack of customizability for individual health systems and clinicians. We developed a HIT interface with, and for, frontline clinician users in safety-net clinic. Safety-net clinics often lack the resources for HIT-based interventions, yet these sites might gain the most from such interventions given the clinician’s workload and disease burden. Patients experiencing socioeconomic vulnerabilities tend to have chaotic lives; modest interventions involving reminders and other notifications have the potential to make significant improvements to appointment adherence and thereby therapeutic control. Moreover, technology-enabled panel management, as in our intervention, will help clinicians triage patients according to risk for harm or loss to follow-up and thereby more efficiently allocate limited resources in this setting.

If successful, our study will demonstrate an example of integration of HIT platforms into existing electronic health records to improve patient care in real-world clinical settings. As we designed the intervention to work within the existing clinic personnel and electronic health record, it also promises to be sustainable and adaptable to other clinics within and outside of the network that share a similar data infrastructure, allowing for the retention of customizations made at pilot sites. However, modified panel management workflows will vary from site to site as the ability to tailor the intervention is, in part, what makes it novel. Finally, the intervention facilitates the use of evidence-based dosing algorithms, thereby promoting the adoption of evidence-based practice in anticoagulation clinics—an important strategy to optimize health outcomes at the population level.

## Abbreviations

ADE: adverse drug event
AHRQ: Agency for Healthcare Research and Quality
AVR: atrial valve replacement
DOAC: direct-acting oral anticoagulant
DVT: deep venous thrombosis
EHR: electronic health record
HIT: health information technology
INR: international normalized ratio
LMM: linear mixed model
NIH: National Institutes of Health
MVR: mitral valve replacement
PAH: pulmonary arterial hypertension
PE: pulmonary embolism
SFDPH: San Francisco Department of Public Health
TTR: time in therapeutic range
